# The performance of the EQ-HWB-S as a measure of quality-of-life of caregivers in families that have experienced adverse events

**DOI:** 10.1007/s10198-024-01688-w

**Published:** 2024-04-05

**Authors:** Cate Bailey, Kim Dalziel, Leanne Constable, Nancy J. Devlin, Harriet Hiscock, Helen Skouteris, Tessa Peasgood

**Affiliations:** 1 Melbourne Health Economics, Melbourne School of Population and Global Health, The University of Melbourne, Level 4, 207 Bouverie St, Carlton, VIC 3053 Australia; 2 Health Services and Economics, Murdoch Children’s Research Institute, Melbourne, VIC Australia; 3 Health Services Research Unit, Royal Children’s Hospital, Melbourne, VIC Australia; 4 Department of Paediatrics, The University of Melbourne, Melbourne, VIC Australia; 5 Health and Social Care Unit, School of Public Health and Preventive Medicine, Monash University, Victoria, Australia; 6 Sheffield Centre for Health and Related Research, School of Medicine and Population Health, University of Sheffield, Sheffield, UK

**Keywords:** Psychometric, EQ-HWB, Families, Adverse life experiences, ACE, Quality of life, I1, I18

## Abstract

**Purpose:**

The recently developed EQ Health and Wellbeing Instrument (EQ-HWB) is a broad, generic measure of quality-of-life designed to be suitable for caregivers. The aim of this study was to investigate performance and validity of the 9-item version (EQ-HWB-S) for caregivers where families had experienced adverse-life-events.

**Methods:**

Using survey data from caregivers of children aged 0–8 years attending a community-health centre in 2021–2022, the general performance, feasibility, convergent and known-group validity, responsiveness-to-change, and test–retest reliability of the EQ-HWB-S was assessed. Twelve semi-structured interviews were conducted with survey respondents to assess acceptability and content validity.

**Results:**

The sample included  234 caregivers at baseline (81% female, mean age 36-years, 38% Australian-born) and 190 at 6-months follow-up. Most EQ-HWB-S item responses were evenly spread, except for ‘Mobility’. The instrument showed good convergent validity with psychological distress (Kessler 6 (K6)) and personal-wellbeing (PWI-A) scales. EQ-HWB-S level sum-scores and preference-weighted scores were significantly different in all known-group analyses, in expected directions, and the instrument was responsive to change. For test–retest reliability, Intraclass Correlation Coefficients were excellent and individual item Kappa scores were moderate. The instrument was well received by interviewees who found the questions clear and relevant. The items were appropriate for parents experiencing adversity and carers of children with additional needs.

**Conclusion:**

The EQ-HWB-S appeared valid, responsive to change, feasible, and well accepted by caregivers. By demonstrating the validity of the EQ-HWB-S in this hard-to-reach population of caregivers in families experiencing adverse events, this study adds to existing international evidence supporting its use.

**Supplementary Information:**

The online version contains supplementary material available at 10.1007/s10198-024-01688-w.

## Introduction

Standard health-related quality-of-life (HRQoL) measures used in economic evaluation are validated in the health sector but may not capture important aspects of the quality-of-life of caregivers [1]. There is a growing body of research outlining the need to include caregivers in economic evaluation to account for potential “spillover” effects when making decision on new health technologies [2]. The effects on the health and wellbeing from caregiving could include fatigue, anxiety, and work-related issues, from a range of caring responsibilities such as caring for a child with a health condition to an elderly parent with dementia [3]. NICE has recommended that these types of spillover effects should be included in economic evaluations since 2013 [4].

There is increasing evidence that caregiver effects are measurable [5], but such effects have rarely been included in Technology Appraisals and Highly Specialised Technologies (HST) guidance [6]. When spillover effects are not included there can be a risk that interventions will be undervalued [7]. In this context, the EQ-HWB (EuroQol Health and Wellbeing instrument) was developed as a broad, generic measure of quality-of-life for use in economic evaluation that would be applicable for patients and caregivers across health, social care and public health sectors [8]. The EQ-HWB items were generated through a qualitative analysis of previously existing HRQoL, carer- and social-related instruments, a review of theoretical frameworks and concepts regarding quality of life, and through incorporating the voices of patients, social care users and carers [1, 9]. Face validity of the items was assessed across six countries (Argentina, Australia, China, Germany, the United Kingdom, and the United States of America [10]). Initial psychometric results indicated that the instrument performed well in classical psychometric testing and item response theory models [8, 9, 11].

Evidence of the validity of the EQ-HWB-S in caregivers has only recently been emerging. Both the EQ-5D and EQ-HWB-S were shown to be able distinguish between participants with and without both mental- and physical-health issues in a recent study [12]. Only the EQ-HWB-S, though, could distinguish between those who were and were not caregivers, and between caregivers with a higher or lower caregiving burden. Results from a recent conference abstract [13] found that the EQ-HWB-S had better discrimination for caregiver status than the EQ-5D and the Adult Social Care Outcomes Toolkit (ASCOT) [14]. Whilst this preliminary evidence is building, there are currently no studies specific to caregiving of parents of younger children or for families experiencing adversity. There is now a need to provide more robust and in-depth validity studies on the EQ-HWB in a wide range of caregiver populations.

The EQ-HWB has two versions, the 25-item full version (EQ-HWB) and the nine-item short form (EQ-HWB-S). Both versions currently have experimental status as additional evidence is generated on the instruments’ validity. This evidence generation includes testing the performance of the EQ-HWB in caregiver populations. The 9-item EQ-HWB-S was chosen for inclusion in the current study for several reasons: there was limited space available in the survey pack for the main study for a longer instrument, the EQ-HWB-S is more likely to be used in economic analysis, and because we expected that preference-weights would soon be available for the EQ-HWB-S but not the EQ-HWB [15].

In this study, we used a mixed-methods approach to investigate the validity of the EQ-HWB-S in a population of caregivers of children aged 8 years and under. The age range of 0–8 years was chosen pragmatically as this is the age group that tends to be cared for by community health organisations in this location. Our first aim was to investigate the general performance, feasibility, convergent and known-group validity, and responsiveness to change of the EQ-HWB-S through survey data of caregivers where families had experienced adverse life events. Our second aim was to use semi-structured, in-depth interviews to investigate acceptability and content validity, including on the 16 items not included in the short form. Finally, we aimed to investigate test–retest reliability on a smaller sample that included the interview participants.

## Methods

### Study design

This study used a mixed-methods design incorporating survey data and semi-structured interviews. The study was nested within a larger evaluation of an integrated Hub model of care in community health services in a low socio-economic area [16]. Health services at the Hub included general practitioners, paediatricians, allied health professionals, maternal and child health nurses as well as social services provided by lawyers, social workers, a financial counsellor and a care navigator to aid families in finding and accessing services. A survey was designed for the overall Hub evaluation that included a range of measures including the identification of adverse life events, referrals to address adversity, and a range of instruments that included the EQ-HWB-S as a measure of quality-of-life for caregivers.

Researchers recruited participants from waiting rooms or through Hub practitioners with permission from clients to be contacted by the researchers. Outcomes were reported by caregivers with a range of complex life circumstances in surveys at baseline and 6-months follow-up. Following the administration of the baseline survey, semi-structured interviews were conducted by the first author with survey participants who had indicated that they were willing to be contacted for further research.

### Adverse life events

Adverse life events were described in the survey as ‘life challenges’ and included events outside of the family (lacking social support, issues with finances, housing and/or employment), inside the family (issues with family physical health, parent mental health, parenting, child neglect, alcohol and substance abuse, family relationships, family violence, child abuse), and broader social needs (issues with visa and immigration issues, crime issues, discrimination) (see Table S1 for full list).

### Study population/participants

The community health Hub was in a low socioeconomic area in the South-Western suburbs of Melbourne.Services were provided to families at low or no cost due to there being clusters of adverse childhood events (ACEs) in families in the area. The population in this area is culturally-diverse; more than 50% of children aged 0–4 years have two parents born outside Australia [17]. Estimates from the Australian Early Development Census indicate that around 23% of children starting school in this area were at risk in at least one developmental domain [17]. Inclusion criteria for caregivers was that they were caring for a child aged between 0 and 8, including pregnant women, and had accessed any service provided through the Hub.

### Procedure

Ethics approval was received from the Royal Children’s Hospital Ethics Committee (HREC/62866/RCHM-2020). Participants received a AUD25 honorarium for each completed survey. Most participants answered questions online, with a small proportion (n = 8, 3.4%) completing the survey by phone with a researcher. Baseline and follow-up surveys were the same. The survey was expected to take approximately 20 min to complete online, or somewhat longer for participants who needed extra support or an interpreter to complete the survey in person or by phone.

For the interviews, a semi-structured interview protocol was developed by the first and senior authors to explore comprehension comprehensibility and relevance [18] of the EQ-HWB-S for this population. The interview was divided into four sections. Participants were asked: (1) what thoughts or words came to mind in regards to their own quality-of-life or wellbeing and the impact of parenthood on this; (2) to describe their thought processes as they answered each of the EQ-HWB-S questions and whether any words or questions were difficult to read or understand, with probing questions from the interviewer; (3) whether they thought that the EQ-HWB-S questions covered the aspects they mentioned in Sect. 1 and (4) the relevance of the 16 EQ-HWB questions not included in the short form of the instrument.

Twelve semi-structured interviews were conducted with survey participants who were purposefully sampled as having more adverse life events (as measured from the survey questions). Interviews were conducted until adequate coverage for adversity intensity was achieved as per recommendations contained in Vasileiou et al. for data adequacy [19]. Data adequacy was expressed here as covering different types and number of adverse events and by ensuring that participants with more adverse events in the survey were included in the interviews. Interviews were conducted one-on-one via zoom or phone by the first author, and participants received an AUD45 honorarium for attending the interview.

### Materials

Instruments in the survey included the EQ-HWB-S, the Personal Wellbeing Index-Adult (PWI-A), the Kessler 6 (K6) and a single-item global health question. The EQ-HWB-S includes 9-items: difficulties getting around inside and outside (mobility), difficulties doing day-to-day activities (activities), feeling exhausted (exhaustion), feeling lonely (loneliness), having trouble concentrating or thinking clearly (cognition), feeling anxious (anxiety), feeling sad or depressed (sad/depressed), feeling like one has no control over day-to-day life (control) and how much pain was experienced (pain) over the last seven days [20]. The PWI-A measures satisfaction with life over seven domains: standard-of-living, health, achievement, relationships, safety, community-connectedness, and future security [21]. The K6 [22] is a commonly used instrument to measure mental health in the general population [23] that screens for mental illness using 6 items: felt nervous, hopeless, restless or fidgety, depressed, everything was an effort, and felt worthless. There are two published sets of cut points for the K6 to identify levels of mental distress. Kessler et al. [24] define two groups as ‘probable’ versus ‘no probable’ mental distress. Prochaska et al. [23] define three groups as ‘serious’, ‘moderate’ and ‘no probable’ mental distress. The ‘probable’ mental distress group as defined by Kessler et al. has the same parameters as the ‘serious mental distress’ groups defined by Prochaska et al. The SF12 global health question is a single question: “In general, would you say your own health is: Excellent, Very good, Good, Fair, Poor?” [25] The number of adverse life events experienced by participants were coded into three groups: 0–1, 2–4, and 5–13 adverse life events.

To calculate child social-emotion symptoms, we used established and standard, validated cut points from the full Ages and Stages Questionnaire if the study child was aged 0–2 years [26], and the age appropriate complete Strengths and Difficulties Questionnaire if the study child was aged 2–8 years [27] (measuring aspects such as communication, emotional symptoms, conduct issues, problem solving and prosocial behaviour). We created a single variable by combining the dichotomous variables for the two age subgroups. Child disability was measured by the question: “Do you have any child with a disability?** (**Disabilities might include sensory, disabilities affecting a child’s hearing or vision, physical disabilities affecting a child’s physical capacity and/or mobility, intellectual disabilities affecting a child’s ability to learn, communicate or retain information, or psychosocial disabilities where a child’s mental health affects social inclusion)” [16]. SEIFA measures socio advantage and disadvantage by postcode [28].

### Statistical analysis

All analyses were performed in STATA version 15. Preference-weights from a pilot UK value-set were applied to produce index-scores for the EQ-HWB-S [15]. The pilot value-set is the first set produced for the EQ-HWB-S. Investigating the psychometric properties of the EQ-HWB-S using the value-set is useful to see how well the scale performs when preference weights are applied. EQ-HWB-S level sum-scores (referred to as sum-scores) were calculated by summing the EQ-HWB-S items (marked 1–5). Each of the 9 items has 5 levels, so the minimum score is 5 (representing no problems on each dimension) and the maximum score is 45 (representing the most severe problems on all dimensions). Where possible, psychometric analyses followed the guidance outlined in the technical methods paper from the QUOKKA research group [29] (a protocol developed by a panel of experts for consistency of reporting of psychometric tests for analyses arising from a multi-instrument comparison study).

Baseline characteristics were calculated using number and percentage for each demographic category. Response distribution and feasibility of the EQ-HWB-S was investigated by calculating numbers and percentages of responses to each item, including missing data. Convergent validity was assessed using Spearman correlations for ordinal data for the EQ-HWB-S against the K6 and the PWI-A for items, sum-scores, and index-scores. We defined correlation strength as per Cohen 1992 [30]; a correlation of 0.1–0.29 is considered weak, 0.3–0.49 moderate, and = > 0.5 strong. Prior to analysis, we hypothesised the correlations that we expected to be moderate (0.3) or above. We did not hypothesise prior to analysis whether the K6 or the PWI-A would have higher correlations with the EQ-HWB-S, as although the PWI-A specifically measured wellbeing, some of the PWI-A items were not expected to correlate highly (such as safety, community-connectedness and future security) with EQ-HWB-S items which aim to capture health-, carer and social care-related quality of life. Known-group validity was assessed using independent *t*-tests where there were two groups (the study child had a disability, caregiver experiencing probable mental distress (K6-two groups), and child social-emotional symptoms above established cut-point) and one-way ANOVAs for comparisons across three groups (K6-three groups, PWI-A, and adverse life events) for EQ-HWB-S sum- and index-scores. We hypothesised that EQ-HWB-S sum-scores would be higher (indicating lower quality-of-life) for caregivers with a child with a disability or with social-emotional symptoms, caregiver probable mental distress (K6) or lower personal wellbeing (PWI-A), and with more adverse life events. Higher index-scores indicate higher quality of life, so we hypothesise that there is a negative relationship between the index score and each of these variables as expected (ie reversed to the sum-score). We used Cohen’s *d* to compare effect sizes for the *t*-tests. Cohen’s* d* effect sizes of 0.2–0.49 were considered small, 0.5–0.79 moderate, and ≥ 0.8 large [30].

Responsiveness to change over time was explored by calculating a change score between baseline and 6-months follow up for the EQ-HWB-S (6-months follow-up minus baseline), such that a negative EQ-HWB-S change in the sum-score would indicate an improvement in quality-of-life, and a positive change score a reduction in quality-of-life. This is reversed for the index scores where a positive EQ-HWB-S index change score would indicate an improvement in quality-of-life, and a negative change score, a reduction in quality of life. Change scores were then calculated for K6, PWI-A, global health (SF12), and number of adverse life events, by categorising these variables into three groups: lowered, the same, and increased (for the categorisation coding, see Table S2a). One-way ANOVAs were used to measure differences between groups for change in EQ-HWB-S scores.

Test–retest reliability was measured two days apart for 25 participants. The two day interval was chosen to reduce the risk of potential changes in health or wellbeing between the two time points, and as per the QUOKKA protocol [29]. We had planned to complete 25 interviews; however, after conducting 12 interviews we had reached adequate data coverage [19]. The questionnaire was sent to the 12 interview participants 2 days before and on the interview day prior to the interview starting. A further 13 participants were recruited who only completed the test–retest data for the EQ-HWB-S. Intraclass Correlation Coefficients (ICC) and their confidence intervals, with a mean rating, absolute-agreement, two-way mixed-effects model for the EQ-HWB-S sum-score as recommended by Koo et al. [31], was used to assess the degree of relatedness between the two time points. We used percentage agreement [32] and Kappa scores [33] to measure the agreement between responses for the individual items. Values with an ICC of 0–0.39 were considered poor, 0.40–0.59 fair, 0.60–0.74 good, and above 0.75 excellent [34]. Weighted Kappa coefficients of 0 to 0.2 were considered poor, 0.21–0.40 fair, 0.41–0.6 moderate, 0.61–0.80 substantial and 0.81 indicated almost perfect agreement [33]. Given there was only two days between each time point, we might expected there would be good to excellent agreement; however, the sample size of 25 is considered inadequate to measure test–retest reliability (see Jones et al. [29], Sect. 14.10).

### Qualitative analysis

Interviews were recorded using Zoom software or by phone (2 interviews) and transcribed verbatim using the automatic Zoom transcription service or by the researchers (phone interviews). All transcripts were manually checked for accuracy. Demographic information for each participants was sourced from the baseline dataset [35]. Data were anonymised and imported into NVivo 12 for analysis. The semi-structured interviews were analysed in two sections. Firstly, the section of the interview directly focussing on the 9 items of the EQ-HWB-S were investigated using a content analysis approach with a focus on discussion or evidence of relevance and comprehension [36]. Here, the first and last authors developed a coding scheme using an iterative method based on the steps used in framework analysis [37]. We then analysed data on the 16 items from the EQ-HWB not included in the short form.

## Results—survey

Recruitment of 234 participants for the baseline data collection was conducted by researchers at the Wyndham Vale Hub between November 2021 and March 2022. Follow-up data were collected for 190 participants 6-month later (81.2% of baseline participants).. Baseline characteristics of the sample are presented in Table 1. The SEIFA distribution has most participants at the extreme ends of the scale. This reflects the location, where most participants live in areas of high disadvantage, but parts are near the coast and have beach frontage with higher house prices.

**Table 1:** Baseline caregiver characteristics

	Full sample N = 234 # (%)	Present (missing)	Mean (SD, range)
Gender		233 (1)	–
Female	189 (81.1)		
Male	42 (18.0)		
Prefer not to say	2 (0.9)		
Country of birth		233 (1)	–
Australia	88 (37.8)		
India, Pakistan, Bangladesh, Sri Lanka	91 (39.1)		
New Zealand	8 (3.4)		
Other	43 (18.5)		
Not specified	3 (1.3)		
Main language spoken at home		232 (2)	–
English	136 (58.6)		
Hindi	24 (10.3)		
Punjabi	14 (6.0)		
Telugu	8 (3.5)		
Urdu	6 (2.6)		
Gujarati	4 (1.7)		
Karen	4 (1.7)		
Other	36 (15.5)		
Identify as Aboriginal or Torres Strait Islander		233 (1)	–
Aboriginal	6 (2.6)		
Torres Strait Islander	0 (0)		
Both	2 (0.9)		
Neither	255 (96.6)		
Highest education level		233 (1)	–
Bachelor’s degree or above	119 (51.1)		
Diploma or equivalent	46 (19.7)		
High school or equivalent	34 (14.6)		
Below high school completion	34 (14.6)		
SEIFA^a^		216 (18)	–
Lowest 20%	158 (73.1)		
Second lowest 20%	0 (0)		
Middle 20%	0 (0)		
Second highest 20%	19 (8.8)		
Highest 20%	39 (18.1)		
Other adults in household		234 (0)	–
No other adults	30 (12.8)		
One or more other adults	204 (87.2)		
Number of children in household		234 (0)	–
0	2 (0.85)^b^		
1	68 (29.06)		
2	101 (43.16)		
3	40 (17.09)		
4 or more	23 (9.83)		
Child with disability		231 (3)	-
Yes	151 (65.4)		
No	80 (34.6)		
Parent age in years	–	229 (5)	35.82 (6.49, 19–66)
Parent Health Status (SF12)	–	234 (0)	2.43 (0.98, 1–5)

^a^SEIFA Quintiles of IRSAD SEIFA ranked by state [28]

^b^Pregnancy was an inclusion criterion for participant recruitment

### Response distribution and feasibility

**Fig. 1:**
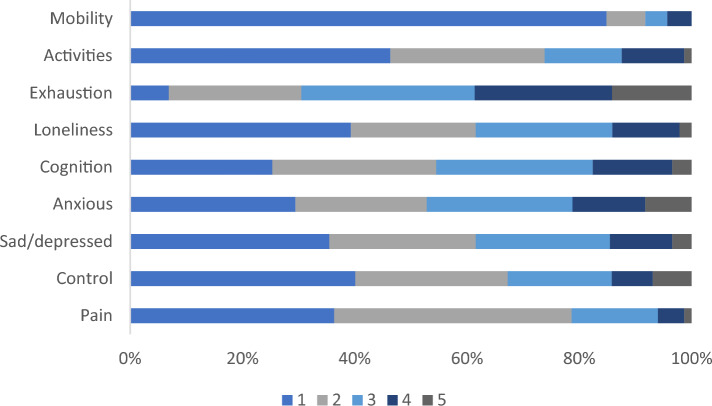
Percentage of responses by EQ-HWB-S items over 5 levels where higher scores indicate lower quality-of-life

Participants were not forced to answer questions to proceed with the survey. Levels of missing data in the baseline EQ-HWB-S results were very low (0–1.3%) with no apparent pattern. As expected, mobility (Item-1), activities (Item-2) and pain (Item-9) were more skewed than items 3–8 (exhaustion, loneliness, cognition, anxiety, sad/depressed, control), which had a more even spread of responses. The mobility item (Item-1) was highly skewed; 84% of participants had no difficulty with their mobility. Participants had high exhaustion scores (Item-3), with only 7% stating that they were exhausted ‘none of the time’, and 14% ‘most or all of the time’. Numbers and percentages of scores by item number are shown in Table 2, and pictorially in Fig. 1. The distribution of EQ-HWB-S sum-scores is shown in Figure S1 and index-scores in Figure S2. The sum- and index-scores correlate at -0.953.

**Table 2:** Distribution of EQ-HWB- S item scores

Item	#(%)	#(%)	#(%)	#(%)	#(%)	#(%)
	No difficulty	Slight difficulty	Some difficulty	A lot of difficulty	Unable	Missing
Mobility	196 (83.8)	16 (6.8)	9 (3.8)	10 (4.3)	0 (0)	3 (1.3)
Activities	108 (46.2)	64 (27.4)	32 (13.7)	26 (11.1)	3 (1.3)	1 (0.4)

	None of the time	Only occasionally	Sometimes	Often	Most/all of the time	Missing
Exhaustion	16 (6.8)	55 (23.5)	72 (30.8)	57 (24.4)	33 (14.1)	1 (0.4)
Loneliness	92 (39.3)	52 (22.2)	57 (24.4)	28 (12.0)	5 (2.1)	0 (0)
Cognition	59 (25.2)	68 (29.1)	65 (27.8)	33 (14.1)	8 (3.4)	1 (0.4)
Anxiety	68 (29.1)	54 (23.1)	60 (25.6)	30 (12.8)	19 (8.1)	3 (1.3)
Sad/depression	83 (35.5)	61 (26.1)	56 (23.9)	26 (11.1)	8 (3.4)	0 (0)
Control	93 (39.7)	63 (26.9)	43 (18.4)	17 (7.3)	16 (6.8)	2 (0.9)

	No physical pain	Mild physical pain	Mod physical pain	Severe physical pain	Very severe physical pain	Missing
Pain	85(36.3)	99 (42.3)	36 (15.4)	11 (4.7)	3 (1.3)	0 (0)

### Convergent validity (concurrent validity)

Convergent validity was assessed using Spearman correlations between the EQ-HWB-S and the PWI-A and K6 questionnaire items and total scores. Correlations in Tables 3 and 4 that are bolded are those that we hypothesised to be at least moderately correlated (at or above 0.3). Of the 63 correlations between EQ-HWB-S and PWI-A individual items, 23 (36.5%) were under 0.3, 40 (63.5%) were over 0.3, and there were no correlations over 0.5, as shown in Table 3. Mobility (Item-1), activities (Item-2) and pain (Item-9) did not correlate highly with the PWI-A items. The correlation between the EQ-HWB-S sum-score and PWI-A total score was over 0.6, and between the EQ-HWB-S index-score and the PWI-A total score almost 0.6. All except two hypothesised item level correlations were at least moderately correlated (over 0.3); we hypothesised that mobility (Item-1) and activities (Item-2) would be related to the PWI-A 2-Health question, but neither was significant in the analysis.

**Table 3:**
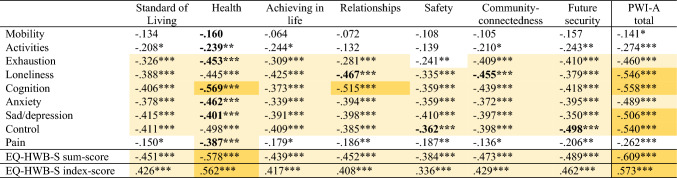
Spearman correlations between EQ-HWB-S and the PWI-A items, sum-scores and index-scores

Hypothesised expected correlations are bolded

*PWI-A* Personal Wellbeing Index-Adult

*p* < 0.05 = *, *p* < 0.01 = **,* p* < 0.001 = ***; white = Rho < 0.3, light yellow Rho 0.3–< 0.5, dark yellow 0.5 < 0.7, gold Rho > = 0.7

**Table 4:**
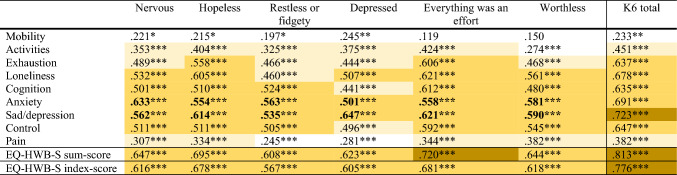
Spearman correlations between EQ-HWB-S and the K6 items, sum-scores and index-scores

Hypothesised expected correlations are bolded

*K6* Kessler 6

*p* < 0.05 = *, *p* < 0.01 = **,* p* < 0.001 = ***; white = Rho < 0.3, light yellow Rho 0.3–< 0.5, dark yellow 0.5 < 0.7, gold Rho > = 0.7

Correlations were high between the EQ-HWB-S and K6 individual items, with only 9 (16.7%) of the 54 correlations between the two instruments being below 0.3, 16 (29.6%) having correlations of 0.3–< 0.5, and 29 (53.7%) of 0.5–< 0.7, as shown in Table 4. The correlation between the EQ-HWB-S sum-score and K6 total score was over 0.8, and between the EQ-HWB-S index-score and the PWI-A total score almost 0.8. All hypothesised correlations were at least moderate (over 0.3) in this analysis. In Tables 3 and 4, correlations for EQ-HWB-S index-scores were slightly lower than EQ-HWB-S sum-scores on all tests.

### Known-group analysis

Mean EQ-HWB-S sum-scores were 19.6 (standard deviation (SD) = 7.0) with scores ranging from 9–44. Preference-weighted scores means were 0.732 (SD = 0.221) and ranged from − 0.313 to 1.0. There were significant differences between groups on *t*-tests for child disability, caregiver mental distress (K6) and child social-emotional symptoms for EQ-HWB-S sum-scores and preference-weighted scores, suggesting that the instrument was able to distinguish between groups, as shown in Table 5. There were large differences in EQ-HWB-S sum-scores and index-scores between mental distress groups (over 10 points on a 45-point scale comparing respondents with probable mental distress to no probable mental distress). Cohen’s *d* scores were large for differences between groups for caregiver mental distress, and moderate for their child’s disability status and their child’s social-emotional symptoms.

**Table 5:** Known-group analysis with mean scores for caregiver EQ-H-WB sum-scores and index-scores by group and *t*-test results

	*n*	Mean (SD)	Mean difference	*t* (*df*)	*p *value	Cohen’s *d*
EQ-HWB-S sum-score						
Caregiver variable						
Caregiver mental distress 2 groups (K6)			10.26	8.79 (216)	< 0.001	1.20
Probable mental distress	32	28.53 (6.95)				
No probable mental distress	186	18.27 (5.95)				
Child variable						
Child disability			4.47	4.75 (221)	< 0.001	0.64
Child with disability	78	22.65 (6.70)				
Child without disability	145	18.18 (6.71)				
Child social-emotional symptoms			5.37	5.95 (214)	< 0.001	0.81
Average to slight	127	17.72 (6.16)				
High	89	23.09 (7.04)				
EQ-HWB-S index-score						
Caregiver variable						
Caregiver mental distress 2 groups (K6)			.339	9.38 (216)	< 0.001	1.38
Probable mental distress	32	.440 (.048)				
No probable mental distress	186	.780 (.171)				
Child variable						
Child disability			0.121	4.03 (221)	< 0.001	0.54
Child with disability	78	0.653 (0.027)				
Child without disability	145	0.775 (0.016)				
Child social-emotional symptoms			0.157	5.43 (214)	< 0.001	0.74
Average to slight	127	0.792 (0.177)				
High	89	0.634 (0.250)				

Effect sizes estimated using Cohen’s* d*. Effect sizes of 0.2–0.49 were considered small, 0.5–0.79 moderate, and ≥ 0.8 large [30]

Child social-emotion symptoms was derived from the Ages and Stages Questionnaire if the study child was aged 0–2 [26], and the Strengths and Difficulties Questionnaire if the study child was aged 2–8 years [27]. Child disability was measured by the question “Do you have any child with a disability? (Further information in “Methods”)

There were significant differences between groups for caregiver mental distress (K6—three groups), the PWI-A (comparing three equal groups) and total adverse life events (0–1, 2–4, 5–13 adverse life events), as shown in Table 6. There were large differences in EQ-HWB-S sum-scores between the upper and lower thirds of the PWI-A groups, of almost 10 points. Post hoc analyses using the Scheffé post hoc criterion for significance indicated that EQ-HWB-S scores were significantly different (*p* < 0.001) between all group combinations for all three tests.

**Table 6:** Known-group analysis with mean scores for EQ-HWB-S sum-scores and index-scores by group, and one-way ANOVA test results

	N	M (SD)	F (*df*)	*p *value
EQ-HWB-S sum-scores				
Mental illness 3 groups (K6)			138.04 (2, 215)	< 0.001
No probable mental distress	94	14.13 (3.75)		
Moderate mental distress	92	22.50 (4.65)		
Serious mental distress	32	28.53 (6.95)		
PWI-A			549.36 (2, 204)	< 0.001
Upper third	72	15.36 (5.23)		
Middle third	72	19.42 (5.48)		
Lower third	63	25.29 (6.71)		
All adverse life events—3 groups			42.45 (2, 203)	< 0.001
0–1 event	42	13.29 (4.20)		
2–4 events	80	18.91 (5.82)		
5–13 events	84	23.76 (6.64)		
EQ-HWB-S index-scores				
Mental illness 3 groups (K6)			105.25 (2, 215)	< 0.001
No probable mental distress	94	0.888 (0.095)		
Moderate mental distress	92	0.669 (0.162)		
Serious mental distress	32	0.440 (0.271)		
PWI-A			36.75 (2, 204)	< 0.001
Upper third	72	0.857 (0.138)		
Middle third	72	0.742 (0.181)		
Lower third	63	0.573 (0.250)		
All adverse life events—3 groups			30.06 (2, 203)	< 0.001
0–1 event	42	0.904 (0.095)		
2–4 events	80	0.758 (0.189)		
5–13 events	84	0.621 (0.237)		

EQ-HWB-S (9-items), *PWI-A* Personal Wellbeing Index-Adult, *K6* Kessler 6

To compare the EQ-HWB-S to the K6 and the PWI-A, we calculated Cohen’s *d* scores for child disability, mental distress, and child social-emotional symptoms. For child disability, the PWI-A had higher Cohen’s *d* scores (0.73) than the EQ-HWB-S (0.64) and the K6 (0.53). For caregiver mental distress, the EQ-HWB-S Cohen’s *d* score (1.20) was higher than the PWI-A (0.83). For child social-emotional symptoms, the EQ-HWB-S Cohen’s *d* score (0.81) was higher than the K6 (0.68) and the PWI-A (0.72).

### Responsiveness to change

There were significant differences in EQ-HWB-S change sum-scores between groups for the K6, the PWI-A, and the global health measure in expected directions, but not for the adverse life events variable. There were significant differences in EQ-HWB-S change index-scores between groups for the K6, and the global health measure in the expected directions, but not for the PWI-A or the adverse life events variable, as presented in Table 7. Post-hoc test results are shown in Table S2b.

**Table 7:** One-way ANOVA results for EQ-HWB-S change score mean (M) and standard deviation (SD) for change reduced, same, or increased scores on the K6, the PWI-A, the global health and the number of adverse life events

	N	Change Mean (SD)	N	Change in mean (SD)	N	Change in mean (SD)	F (*df*)	p
EQ-HWB-S sum-scores^a^								
Change in K6		Reduced mental health		No change		Improved mental health		
	51	2.76 (5.07)	64	− 0.23 (4.02)	59	− 2.25 (5.17)	15.35 (2, 171)	< 0.001
Change in PWI-A^b^		Reduced personal Wellbeing		No change		Improved personal wellbeing		
	82	1.17 (5.59)	40	− 0.75 (4.71)	52	− 1.35 (4.41)	4.46 (2, 171)	0.013
Change in global health		Reduced health		No change		Improved health		
	55	2.24 (517)	95	− 0.26 (4.81)	40	− 3.15 (3.93)	14.94 (2,187)	< 0.001
Change in adverse life events		More events		No change		Less events		
	20	− 0.64 (4.20)	156	− 0.07 (5.15)	14	− 0.40 (5.35)	0.11 (2, 187)	0.898
EQ-HWB Index-scores^b^								
Change in K6		Reduced mental health		No change		Improved mental health		
	51	− 0.081 (0.184)	64	− 0.005 (0.147)	59	0.062 (0.176)	9.79 (2, 171)	< 0.001
Change in PWI-A^b^		Reduced Personal Wellbeing		No change		Improved Personal Wellbeing		
	82	-0.040 (0.202)	40	0.021 (0.129)	52	0.023 (0.159)	2.77 (2, 171)	0.066
Change in global health		Reduced health		No change		Improved health		
	55	− 0.061 (0.178)	95	− 0.006 (0.173)	40	0.094 (0.133)	14.94 (2, 187)	0.013
Change in adverse life events		More events		No change		Less events		
	20	0.006 (0.165)	156	− 0.002 (0.180)	14	0.005 (0.143)	0.03 (2, 187)	0.974

EQ-HWB-S (9-items), *PWI-A* Personal Wellbeing Index-Adult, *K6* Kessler 6; Global health in the SF12 single-item global health question

^a^EQ-HWS change in sum-score was calculated from 6-months follow-up minus baseline scores. Positive mean EQ-HWB-S change sum-scores indicate higher EQ-HWB-S sum-scores at follow-up (reduced quality-of-life). Negative EQ-HWB-S change scores indicate reduced EQ-HWB-S sum-scores at follow-up (improved quality-of-life)

^b^EQ-HWS change in index-score was calculated from 6-months follow-up minus baseline scores. Positive mean EQ-HWB-S change index-scores indicate reduced EQ-HWB-S index-scores at follow-up (improved quality-of-life). Negative EQ-HWB-S change index-scores indicate higher EQ-HWB-S index scores at follow-up (reduced quality-of-life)

## Results—interviews

Baseline characteristics of the 12 interview participants (83% female) are shown in Table S4. Although we sampled specifically for participants with more adverse life events, the interview sample had a higher percentage of participants born in Australia (67%) compared to the baseline sample (38%) and English was the main language spoken at home in 83% rather than 59% of participants in the baseline sample. There were slightly more parents with children with a disability in the interview sample (75%) compared with the baseline sample (65%), and fewer participants with a bachelor’s degree (42%) compared to the baseline sample (51%).

The analysis focused on three broad themes: Interpretation of items (with 4 subthemes identified), Relevance for specific populations (with 3 subthemes identified), and Inclusion of other items into the short form (no subthemes). Only one participant commented on the response wording, and this was positive: “*I like that you have actually used wording and said: ‘more often’, ‘sometimes’[*etc*.], because a one to 10 scale, really, it just doesn't work when it's not explained properly*.” (P1).

### THEME 1—interpretation of items

**Understanding** Participants mostly stated that they found the questions clear. One participant said that they had to read the activities item (Item-2) twice, but still said that they found it clear: “*I just read it over twice and went okay, there's no difficulty*.” [P3] Questions 3–8 (exhausted, lonely, concentration, anxiety, sad/depressed and lack of control) were particularly well received by participants, being short and easy to read.

**Ambiguity of interpretation** The item most open to interpretation was activities (Item-2). Participants described various interpretations of this item, including whether it referred to mental or physical issues or time constraints, “*Some people would be thinking… what context do you mean? Mentally, sometimes I can’t leave the house. Was it your mental health or physical pain, or time?*” (P4). Other interpretations included lack of time due to attending multiple appointments for children with special needs (P5), caring for particular or multiple children, “*my daughter [is] autistic, I can’t take her to the shops, point blank. Taking all six of the kids that are in my care to the shops is very difficult*,” (P4), lack of transportation “*I don’t think this question is relevant to me because I don’t drive*” (P9), or difficulty in completing housework tasks due to issues with their own mental health condition. (P1). For the loneliness question (Item-4), one participant suggested that including an example would make it clearer whether the question meant the ‘feeling of being lonely’ or whether one had people around (P10).

Some participants found that they changed their responses on reflection, suggesting some ambiguity in responses. For instance: “*Now that I’m actually verbalising these questions, I feel like my answers are really different* (P1)”. For activities (Item-2), a participant stated that: “*The first time I tick ‘no difficulty’ because I am able to do things, even though it is a bit difficult…. this morning I tick ‘slight difficulty’…. I can either ‘slight difficulty’ or ‘some difficulty’ in my situation*” (P10), as they factored in being a parent of a child requiring the extensive 24-h caregiving that they share with their partner.

**Use of question examples as an interpretation aid** Despite prompting from the interviewer, participants made few comments relating to the use of examples to illustrate the instrument questions, and these comments were mostly reinforcing that the examples helped with interpretation. For mobility (Item-1), one participant used the example (“using e.g. a walking stick or wheelchair if you normally use them”) to make their response: *“…with an example like that…I [see it is] not relevant to me, so I tick ‘no difficulty’*” (P10). The additional explanation included in the control question (Item-8) helped one of the participants understand the question (P12).

**Question order** The questions flowed well in their current order, except for the first two questions. One participant was confused about the relevance of the first item (mobility): “*I was a bit confused with that one to be honest, I wasn’t too sure it was relevant… I was thinking, what ‘did I sign up for’?*” (P4). Another interpreted the activities item (Item-2) by relating it to Item-1: *“…. because I think it is linked to the first question*” (P6). In response to this information, we asked participants in the later interviews whether they felt we should swap the question order. There was moderate agreement that the activities item (Item-2) may work better if it was presented first “*I think [swapping the items] will be a good idea, because [item-2] is shorter [and] easier to comprehend than the first one, so you quickly get your audience. I spent quite a bit thinking about the first one. Number two is more relatable”* (P12).

### THEME 2—relevance for specific populations

**Appropriateness of scale for parents of young children** Questions 3–8 (exhausted, lonely, concentration, anxiety, sad/depressed and lack of control) were seen as highly relevant to participants’ life circumstances, and caregivers frequently referred to their roles as parents when explaining why they had made a particular response to an item. This was especially true regarding the exhaustion question (Item-3): “*Yes, always, always exhausted. Most parents are going to say they feel mentally exhausted all the time*” (P4). Lack of control (Item-8) was also an issue for parents: “*As a parent you don't get a choice, usually, in what you do. I don't feel like I have much control over my day-to-day life*” (P1), as was loneliness (Item-4): “*Just being with kids, that can make you feel lonely at times*” (P11). Lack of concentration (Item-5) was also related to parenting: “*Because I’m bombarded, constantly bombarded with things… not so much at work, but at home,*” (P12) as was anxiety (Item-6): “*the anxiety is always around the kids, so I am always a bit on the edge a little bit*” (P12).

The mobility question (Item-1) was seen as the least relevant item in this population, as few participants had mobility issues; however, participants felt that the item was still important to retain for other people. Participants generally had only moderate issues with pain (Item-9), but also felt that the question was important to keep for other people “*pain can really impact someone's quality of life*” (P8).

Participants described how their answers would have been different at various times, suggesting that the instrument was able to detect changes in quality-of-life at different times for parents like them. For instance, on the depression item (Item-7), one participant said: “*I mean, depression always rears its ugly head, but I find that I can control it a bit better these days*.” (P1). Another participant described how their answers were different than usual due to an acute health condition: *“I was having trouble concentrating… I’m usually very clear*.” (P2). Here, the participant had accurately remembered the recall period of 7 days.

**Appropriateness in an adversity setting** The questions resonated well with participants experiencing adverse life events. Questions were highly pertinent for a participant with many children under her care and custody issues with child protection, and another participant who had experienced domestic violence. Items for lack of control, concentration and pain were particularly pertinent to these two women: “*I still suffer a lot of PTSD Symptoms…from the domestic violence and the constant stress”* (P3, re pain; Item-9). “*I don't have that control—everything else is around me is controlling me somehow*” (P5, re ‘Control’).

**Appropriateness for carers of a children with additional needs** The instrument seemed particularly appropriate for carers of children with special needs, such as physical disabilities and diagnosis of Autism Spectrum Disorder. Participants cited a lack of time for close relationships and lack of belonging (loneliness, Item-4), constant demands on time and restriction on activities including work (activities, Item-2), exhaustion (Item-3, almost all participants), lack of control (Item-8): *“I have applied for the [disability pension] and got rejected and I’m still going through the diagnosis for my son's autism, which will allow funding [for] support in the classroom. So, I don't have those controls over my life that I wish I had*” (P3) and cognition “*…I really struggle with concentrating… it's gotten worse since I’ve had kids, but I think because there's so much on my mind, I just get distracted”* (P1).

### THEME 3—inclusion of other items to the short form

**Inclusion of items in the instrument** We asked participants whether any of the 16 items not included in the short form should have been included. For most of these items there was a varied response, except for the “sleep” item. Eleven of the 12 participants felt the sleep should be included in the short form, and many participants were quite adamant about this: “*I think [there should be] a separate [item] for sleep because sleep is so, so vital and so many parents don't get enough of it*,” (P1) and “*Yeah, this one is a definite. This has to be there in the questions*” (P11). Only one participant (P12) felt that it could be omitted: “*because you already covered that … in day-to-day work*.” Participants also mentioned the impact on finances from caregiving as a factor that was not included in either of the EQ-HWB versions: “*A lot of my day is dictated by constraints that already exist in my life like having a child or financial constraints”* (P8).

## Results test–retest reliability

For the full dataset of 25 participants, the ICC was 0.87 (95% CI 0.69–0.94, F(24, 24) = 7.28, p < 0.001), which is considered excellent[34]. We identified five participants who did not conform to the study protocol as they had completed the interview between the two tests. We repeated the analysis without these five cases, in case doing the interview had affected participant’s perception of the instrument items. For the reduced sample of 20 participants, the ICC was almost the same at 0.87 (95% CI: 0.69–0.95, F(19, 19) = 7.92, p < 0.001), indicating some stability in the data despite the inadequate sample size.

For the individual items, percentage agreement scores and Kappa scores were calculated. For all items, there was percentage agreement above 80% in the full dataset (*n* = 25) and the reduced dataset (*n* = 20). Kappa scores ranged from 0.38 to 0.61 in the full dataset, and from 0.41 to 0.59 in the reduced dataset, suggesting moderate agreement [33]. Percentage agreement and Kappa scores are presented in Table S5a and Table S5b in the supplementary files.

## Discussion

In this study, we tested the use of the EQ-HWB-S for caregivers of young children where families had experienced adverse life events. Using a mixed-methods design has allowed us to benefit from a deeper and contextualised understanding from the qualitative data to the more generalisable results from the quantitative data[38]. Through analysis of survey results, we found that the instrument was feasible, showed strong convergent validity (both with a validated measure of psychological distress and with a measure of personal wellbeing based on satisfaction across seven life domains), strong known-group validity (including known groups based on proxies for carer burden), and was responsive to change. Data from interviews with participants indicated that the instrument was well received and had good content validity. In known-group analysis, the EQ-HWB out-performed the K6 and the PWI-S when considering effect size. The EQ-HWB-S appeared to be suitable for parents, in an adversity setting, and for carers of children with additional needs. There was moderate to excellent test–retest reliability despite the inadequate sample size.

The qualitative results gave context to quantitative results. In the interviews, participants found items 3–8 (exhausted, lonely, concentration, anxiety, sad/depressed and lack of control) particularly easy to understand and answer, and we note that these items had a good spread across responses in the survey items. There were few participants with mobility issues (Item-1), as could be expected in this population. Despite this, interview participants felt that the item was important to retain for other people in other contexts, such as older people. Having a question that was not personally relevant at the start of the instrument was seen as problematic by some caregivers, who felt that the activities question may work better as a first item.

We found high levels of exhaustion in this population of caregivers where families had experienced adverse life events, in the survey data. Through the interviews, we saw that exhaustion was linked to parenting, especially for parents of children with health conditions or a disability. We find similar outcomes in previous research on parents of children with health conditions [39, 40], and this was explored in depth in an Italian cohort of parents during a COVID-19 lockdown, where participants experienced high levels of exhaustion from parenting, and particularly so when parenting a child with special needs, younger aged children, and when single parenting [41]. In the interviews, some caregivers found it difficult to decide on a correct response for the activities item because their response varied depending upon whether they were thinking about completing activities with or without the presence of children. When asked about items included in the long form of the EQ-HWB but not the short-form, sleep was considered important to include by almost all interview participants. Sleep was most often seen as distinct from exhaustion, and impacted on by caring for children, and especially children with health conditions. The impact on finances from caregiving was raised in some interviews as being an important factor; such an item is not included in either the long or short version of the instrument.

In the survey data, the instrument discriminated well between known groups, with significant mean differences between all groups for EQ-HWB-S scores. When comparing known-group effect sizes between the EQ-HWB-S, the K6 and the PWI-A, the EQ-HWB-S had the highest effect sizes in 2 of the 3 variables, suggesting that the EQ-HWB-S may have higher discriminant ability than the K6 or the PWI-A. There were high correlations between caregiver mental distress (K6) and the EQ-HWB-S items and sum-scores, suggesting that the EQ-HWB-S may be measuring similar constructs of mental wellbeing in this population.

For the responsiveness to change analysis, we note that baseline data were partly collected during the extensive Melbourne COVID-19 lockdowns, which may have led to differences in health and wellbeing across time points. There were significant differences in change over time, in expected directions, for the EQ-HWB-S sum- and index-scores against the K6 and the global health item, but not in the adverse life events item. This finding suggests that the EQ-HWB-S may be better at picking up physical and mental health impacts that result from adversity rather than the number of actual adverse life events experienced. The lack of significant differences between groups in the responsiveness to change analysis for adverse life events may also have been due to the small sample size of participants changing groups in that variable, that the simple count information did not account for the differential impact that some adverse life events might have on family members, or that there were differences in the severity of the adverse life events. Investigating responsiveness to change was out of scope for the qualitative interviews.

We found three overall themes in the semi-structured interview data. In respect to the interpretation of the items (Theme 1), participants generally found the items to be clear and easily understood. The Activities item was the most ambiguous in terms of interpretation, the examples used in some questions were generally found to be useful to help interpretation, and there were some concerns about question order with some participant considering that the mobility (Item 1) and activities (Item 2) could be swapped. We were particularly interested in how well the scale worked in this population (Theme 2). We found that the scale worked well for parents of children in this age range, and specifically that it was well accepted in an adversity setting and where children had additional needs. Our final theme addressed the question of whether the right items from the EQ-HWB had been included in the EQ-HWB-S. As noted above, only the sleep item was strongly endorsed as being important to include in the short form; other items had mixed interpretations between participants.

### Limitations

This is the first study to investigate the psychometric properties of the EQ-HWB-S in an adversity setting and to investigate validity and reliability of the EQ-HWB-S in any sample of caregivers with young children. Strengths of the study include that this was a mixed-methods study using a sample with broad socio-economic status and cultural spread. The sample size for the baseline survey of 234 participant caregivers was reasonable for such a hard-to-reach sample. The test–retest sample, at 25 cases, was likely too small to achieve accurate Kappa scores, but gave a general indication of reliability that can be built on in future studies. There were five test–retest cases which were not completed according to the protocol; however, removing these cases did not significantly change the ICC or Kappa scores.

The baseline and follow-up surveys supplied rich data to investigate the psychometric properties of the EQ-HWB-S in caregivers of children where families had experienced adverse life events with added context from the qualitative interviews. For the survey data, these families included participants who required translators which were sourced at the community health Hub; for the qualitative study, only participants who spoke fluent English were invited to participate, due to lack of access to interviewers who were fluent in other languages for this part of the study. We did not include responsiveness to the Hub intervention in the responsiveness to change analysis, as implementation was only in its early stages at six-months follow-up. Using the level sum-score has limitations, as the same sum-score can have very different profiles, and giving equal weight to dimension makes assumptions about their relative importance[42]. Only pilot index weights for the UK were available for the EQ-HWB-S[15]; future studies will be strengthened as more country-specific weights become available. We note that the population was generally well-educated, with only 15% not having completed schooling. As the population had a high migrant component, it is possible that, although well-educated and thus able to migrate, many migrants may not have comparable work to others in Australia with the same education level. We were unable to compare outcomes between male and female caregivers in any analyses due to the small and uneven sample size. Recent work has found preliminary evidence comparing the psychometric performance of the EQ-HWB-S to other carer quality-of-life instruments (such as the CarerQoL [43], the ASCOT [14] or the Carer Experience Scale [44]) in residents in aged care [45]; this type of study would be a logical next step for validating the EQ-HWB-S in caregivers.

## Conclusions

The EQ-HWB-S showed validity, was sensitive to change, feasible and well accepted by caregivers in this population. Our findings support the very limited data from previous studies that the EQ-HWB-S shows validity in child caregiver populations. The study included participants that can be challenging to reach, making this paper a valuable contribution to the evidence supporting the use of the EQ-HWB-S for caregivers and in an adversity setting. We are still in the early stages of validating the EQ-HWB-S as a suitable tool for measuring caregiver quality-of-life for economic evaluation. Further research is now required to confirm these results in similar cohorts, and to investigate the use of the EQ-HWB-S in other caregiver groups.

## Supplementary Information

Below is the link to the electronic supplementary material.

Supplementary file1 (DOCX 60 KB)

## Data Availability

Data is not available due to ethics approval restrictions.
