# Supplementary material for: The performance of the EQ-HWB-S as a measure of quality-of-life of caregivers in families that have experienced adverse events
Source: Eur J Health Econ. 2024 Apr 5;26(1):7–21. doi: 10.1007/s10198-024-01688-w (PMC11743351; doi:10.1007/s10198-024-01688-w)
Supplement: Supplementary file 1 — Supplementary file1 (DOCX 60 KB) [file 10198_2024_1688_MOESM1_ESM.docx]

# The performance of the EQ-HWB-S as a measure of quality-of-life of caregivers in families that have experienced adverse events.

# Supplementary figures

*Figure S1* Distribution of EQ-HWB-S sum-scores

*Figure S2* Distribution of EQ-HWB-S index-scores

# Supplementary tables

#### Table S1

**Adverse life events (life challenges) included the survey**

1. Not enough contact with or support from others for yourself
2. Not enough money for everyday things such as food, clothing or bills
3. Problems with housing like worrying about keeping your home, having to share your home, or having a house that's too crowded, or in need of repair
4. Someone in the family having problems finding or keeping a job, insecure employment or a job that is not family friendly
5. My own physical health or disability or that of another family member inside or outside of my home
6. My own challenging feelings like feeling emotional, depressed, angry, anxious, exhausted or even having strange thoughts such as harming myself or others
7. The way that I (or my partner) manage my child/ren's daily routines, physical needs and their behaviour
8. My child/ren being left to look after themselves too much, not having their needs met, or being given too much responsibility for their age
9. Someone in my family drinking alcohol or using drugs
10. Conflict or tension between members of my family
11. My child/ren might be seeing or exposed to behaviour within the family/ at home that frightens them like threats, bullying, yelling, screaming, putting people down, hitting, slapping, kicking, or punching
12. My child/ren is being harmed or hurt by someone
13. Issues with visa or immigration for someone in my family or myself.
14. My own or a family member's court appearances as a defendant, being on bail, parole or spending time in prison.
15. Someone in my family has experienced discrimination or harassment such as bullying, racism.

#### Table S2a

Change scoring for the K6, PWI-A, global health question, and adverse life event scoring (as used in Table 5).

|  | Reduced | No change | Improved |
| --- | --- | --- | --- |
| K6 score | -2 or less | -1 to 1 | 2 or more |
| PWI-A score | -3 or less | -2 to 2 | 3 or more |
| Global health score | -1 or less | 0 | 1 or more |
| Number of Adverse life events | -1 or less | 0 | 1 or more |

Notes: EQ-HWB-S (9-items), PWI-A = Personal Wellbeing Index-Adult, K6 = Kessler 6; Global health and a single-item global health question.

#### Table S2b

Scheffe Post-hoc tests for one-way ANOVAs for the responsiveness to change analysis, mean (significance)

| **EQ-HWB-S sum-score** | Test 1-2  Mean (*p*-value) | Test 1-3  Mean (*p*-value) | Test 2-3  Mean (*p*-value) |
| --- | --- | --- | --- |
| K6 | 3.0 (.003) | 5.0 (<.001) | 3.0 (.063) |
| PWI-A | -.60 (1.00) | -2.52 (.020) | -1.92 (.151) |
| Global health | 2.50 (.007) | 5.39 (<.001) | 2.89 (.004) |
| Adverse life events | n/a | n/a | n/a |
| **EQ-HWB-S index-score** |  |  |  |
| K6 | .076 (.054) | -.143 (<.001) | .067 (.087) |
| PWI-A | -.003 (.998) | -.064 (.126) | -.061 (.198) |
| Global health | -.054 (.168) | -.155 (<.001) | .100 (.005) |
| Adverse life events | n/a | n/a | n/a |

Notes: EQ-HWB-S (9-items), PWI-A = Personal Wellbeing Index-Adult, K6 = Kessler 6; Global health and a single-item global health question.

#### Table S4

Baseline characteristics of the interview sample, N=12

|  | #(%) |
| --- | --- |
| Female | 10 (83) |
| Country of birth is Australia | 8 (67) |
| English main language spoken at home | 10 (83) |
| Identify as Aboriginal and/or Torres Strait Islander | 1 (8) |
| Bachelor’s degree or above | 5 (42) |
| SEIFA quintiles |  |
| Lowest | 5 (46) |
|  | 0 (0) |
|  | 0 (0) |
|  | 2 (18) |
| Highest | 4 (36) |
| Number of children in household |  |
| 0-1 | 4 (33) |
| 2-3 | 5 (42) |
| 4 + | 2 (25) |
| Child has a disability | 9 (75) |

#### Table S5a

Test-retest reliability analysis, percentage agreement and Kappa scores for full dataset (n=25)

|  | Agreement % | Expected agreement % | Kappa | SE | z | p |  |
| --- | --- | --- | --- | --- | --- | --- | --- |
| 1 Mobility | 88.00 | 78.88 | 0.43 | 0.158 | 2.74 | 0.003 |  |
| 2 Daily activities | 82.67 | 68.69 | 0.45 | 0.141 | 3.18 | <0.001 |  |
| 3 Exhaustion | 85.00 | 67.88 | 0.53 | 0.132 | 4.05 | <0.001 |  |
| 4 Lonely | 87.00 | 77.40 | 0.43 | 0.139 | 3.06 | 0.001 |  |
| 5 Concentrate | 88.00 | 74.40 | 0.53 | 0.129 | 4.12 | <0.001 |  |
| 6 Anxiety | 85.00 | 66.84 | 0.55 | 0.132 | 4.14 | <0.001 |  |
| 7 Sad/depressed | 90.00 | 75.68 | 0.59 | 0.134 | 4.37 | <0.001 |  |
| 8 Control over life | 84.00 | 74.32 | 0.38 | 0.125 | 3.03 | 0.001 |  |
| 9 Pain | 86.00 | 64.40 | 0.61 | 0.141 | 4.30 | <0.001 |  |

#### Table S5b

Test-retest reliability analysis, percentage agreement and Kappa scores - reduced dataset (n=21)

|  | Agreement % | Expected agreement % | Kappa | SE | z | p |
| --- | --- | --- | --- | --- | --- | --- |
| 1 Mobility | 87.50 | 75.75 | 0.49 | 0.168 | 2.89 | 0.002 |
| 2 Daily activities | 81.67 | 68.67 | 0.41 | 0.156 | 2.66 | 0.004 |
| 3 Exhaustion | 86.25 | 69.25 | 0.55 | 0.144 | 3.84 | <0.001 |
| 4 Lonely | 86.25 | 76.00 | 0.43 | 0.153 | 2.79 | 0.003 |
| 5 Concentrate | 86.25 | 73.00 | 0.49 | 0.143 | 3.42 | <0.001 |
| 6 Anxiety | 86.25 | 66.13 | 0.59 | 0.149 | 3.99 | <0.001 |
| 7 Sad/depressed | 90.00 | 75.50 | 0.59 | 0.152 | 3.90 | <0.001 |
| 8 Control over life | 83.75 | 74.13 | 0.37 | 0.139 | 2.68 | 0.004 |
| 9 Pain | 82.50 | 67.00 | 0.47 | 0.155 | 3.03 | 0.001 |
